# Announcement

**Published:** 2015-03-20

## World Water Day — March 22, 2015

World Water Day, sponsored by the United Nations, is observed every year on March 22. This year, World Water Day focuses on water and sustainable development. Water is a finite resource that is fundamental to human health and well-being. However, an estimated 700 million persons lack access to improved sources of drinking water and 2.5 billion persons are without improved sanitation facilities,* putting them at risk for illness or death (1).

Water also plays a vital role in strengthening the resilience of social, economic, and environmental systems in the face of rapid and unpredictable changes. From food and energy security to human and environmental health, water is an essential part of sustainable development. As cities around the world, particularly in developing countries, continue to grow at an exponential rate, global water use is projected to increase by 55% through 2050, because of growing demands from manufacturing, thermal electricity generation and domestic use (2). Managing water resources efficiently and responsibly in the face of this growth and committing to developing a deeper understanding of the interconnections between water, food, energy, health, trade, and the environment are vital to ensuring sustainable development.

Additional information about World Water Day and ideas on how to get involved are available at http://www.unwater.org/worldwaterday. Information regarding CDC’s efforts to ensure global access to improved water, sanitation, and hygiene resources is available at http://www.cdc.gov/healthywater/global.

## Footnotes

* An improved water source is defined as water that is supplied through a household connection, public standpipe, borehole well, protected dug well, protected spring, or rainwater collection. Improved sanitation facilities usually ensure separation of human waste from human contact.

## References

[1] United Nations Children’s Fund, World Health Organization (2014). Progress on drinking water and sanitation: 2014 update.

[2] WWAP (United Nations World Water Assessment Programme) (2014). United Nations World Water Development Report 2014, water and energy.

